# Alcohol and the aging cardiovascular system: a dangerous synergy uncovered

**DOI:** 10.1007/s11357-025-01644-3

**Published:** 2025-04-22

**Authors:** Csaba Szabo

**Affiliations:** Section of Pharmacology, Department of Oncology, Microbiology and Immunology, Faculty of Science and Medicine, University of Fribourg, Fribourg, Switzerland

Excessive alcohol consumption heightens the cardiovascular risks by causing hypertension, stroke, arrhythmias, coronary artery disease, cardiomyopathy, and sudden cardiac death [1–3]. As global populations age and alcohol consumption among older adults rises, understanding the intersection between chronic alcohol exposure and age-related cardiovascular decline becomes increasingly urgent [4].

In their comprehensive study, Mukhopadhyay et al. [5] deliver a critical advance in this area by demonstrating that chronic alcohol consumption not only accelerates cardiovascular aging but also significantly impairs cardiovascular reserve capacity, compounding the effects of natural aging. The paper, published in *GeroScience*, represents one of the most detailed preclinical investigations into how long-term alcohol intake disrupts the aging cardiovascular system and contributes to diminished physiological resilience.

The investigators used a 6-month Lieber-DeCarli liquid alcohol diet (5% ethanol) in young (3 months) and aging (24–26 months) male Fisher F344BNF1 rats—an established model for studying cardiovascular aging. This allowed them to dissect both the independent and interactive effects of aging and alcohol on cardiac and vascular structure and function [5]. Their approach is commendably thorough, incorporating, invasive hemodynamics, pressure–volume loop analysis, vascular reactivity assays, histology, immunohistochemistry, and molecular biology to assess mitochondrial function, oxidative/nitrative stress, inflammation, apoptosis, senescence, lipid metabolism, and fibrosis.

One of the central findings is that chronic alcohol exposure impairs mitochondrial function—already a vulnerable target in aging hearts. Specifically, alcohol suppressed the activity of mitochondrial complexes I, II, and IV in both young and aged animals, with the most pronounced dysfunction observed in aged hearts. This mitochondrial impairment was accompanied by marked increases in myocardial and vascular oxidative and nitrative stress, as evidenced by elevated levels of 4-hydroxynonenal (4-HNE) and 3-nitrotyrosine (3-NT), both histologically and via ELISA [5].

Beyond oxidative stress, the study reveals that alcohol acts as a driver of low-degree inflammation, particularly when layered on top of age-related immune changes. Alcohol-fed animals, especially in the aged group, exhibited higher expression of pro-inflammatory cytokines such as TNF-α, IL- 1β, and MIP- 1α, along with increased infiltration of macrophages (F4/80-positive cells) in the myocardium. This inflammatory environment, together with mitochondrial dysfunction and redox imbalance, promotes myocardial apoptosis (via caspase 3/7 activity and DNA fragmentation) and PARP-mediated cell death—both of which were exacerbated in aged, alcohol-fed rats [5].

Notably, cellular senescence was another key pathological feature exacerbated by alcohol in both young and aged rats, with significantly elevated β-galactosidase activity observed in cardiac and vascular tissues [5]. This supports the concept that alcohol acts as a gerotoxin, accelerating biological aging by promoting stress-induced senescence pathways. The link between chronic alcohol intake and premature cardiovascular aging is thus strongly reinforced. Additionally, chronic alcohol consumption, particularly in aging rats, exacerbated disrupted lipid metabolism in aging, as indicated by elevated serum LDL cholesterol levels.

An additional, highly valuable aspect of this study is its high-resolution characterization of cardiovascular function using pressure–volume (PV) loop analysis [6]. This approach is especially valuable in the context of aging and alcohol exposure, which exert complex and interdependent effects on cardiac performance and vascular tone [7–9]. These hemodynamic interactions influence both heart rate and loading conditions, thereby limiting the sensitivity of conventional echocardiography in isolating intrinsic, load-independent measures of myocardial contractility. By utilizing invasive PV analysis, which is widely regarded as the gold standard for in vivo hemodynamic assessment, the authors achieved a precise and comprehensive evaluation of cardiac and vascular function, including bioenergetics [7]. In young rats, alcohol consumption significantly impaired systolic function, as evidenced by reductions in ejection fraction, stroke volume, +dP/dt_max, and stroke work, despite stable heart rates [5]. These deficits, corroborated by various load- and heart rate–dependent indices of myocardial performance, were even more pronounced in aged rats and further exacerbated by alcohol exposure. Diastolic dysfunction was already evident in aged animals, characterized by increased ventricular stiffness, prolonged relaxation times (Tau Weiss and Tau Glantz), and elevated end-diastolic pressure, all accompanied by enhanced fibrotic remodeling [5]. Chronic alcohol intake further worsened each of these parameters in aged rats. In contrast, young alcohol-exposed rats did not exhibit diastolic dysfunction or fibrosis. Collectively, these findings closely resemble the clinical phenotype of heart failure with preserved ejection fraction (HFpEF), a highly prevalent and therapeutically challenging condition in the aging population.

Importantly, the study also offers new mechanistic insights into the deleterious impact of alcohol consumption on the vasculature. Vascular rings from alcohol-fed rats showed impaired endothelium-dependent vasorelaxation, increased reactive oxygen species (ROS) production, lipid peroxidation, and cellular senescence. These effects were especially pronounced in aging animals. Increased total peripheral resistance (TPR), another hallmark of vascular aging, was further elevated by alcohol [5]. Vascular dysfunction contributed to ventriculo-arterial uncoupling—reflected by an increased arterial elastance to end-systolic elastance ratio—ultimately reducing cardiac mechanical efficiency. Arterio-ventricular coupling, the dynamic interplay between the heart and vasculature, is essential for cardiovascular health [10, 11]. Disruption of this balance, common in aging, hypertension, and heart failure, is a strong predictor of morbidity and mortality. Mechanoenergetic efficiency—defined as the ratio of stroke work to pressure–volume area (PVA), and influenced by factors such as end-systolic elastance—is a key measure of cardiac performance, indicating how well the heart converts metabolic energy into mechanical work [12]. This efficiency is often impaired in heart failure, hypertension, ischemic heart disease, aging, and diabetes, correlating with worse outcomes. Chronic alcohol consumption in both young and aging animals caused ventriculo-arterial uncoupling and reduced efficiency, likely driven by mitochondrial dysfunction and oxidative stress. Tracking these changes in individuals with alcohol use disorder may aid in cardiovascular risk assessment and management.

This concept of diminished cardiovascular reserve capacity, brought about by simultaneous dysfunction in both cardiac and vascular systems [5], is a key innovation of the study. The authors articulate this using the framework of mechanoenergetic coupling and reserve, showing that alcohol’s effects are not limited to isolated cardiac damage but instead reflect a system-level degradation of performance, resilience, and adaptability—hallmarks of aging.

From a translational standpoint, the paper’s findings highlight the deleterious aspects of alcohol consumption. Older adults tend to consume alcohol at levels that exceed recommended guidelines, often unaware of the compounding risks posed by their age-related physiological vulnerability [4]. If we apply the conclusions of the preclinical findings reported in this paper to human beings, we can conclude that even in the absence of overt cardiovascular disease, chronic alcohol use may push aging hearts and vessels toward dysfunction and failure by overwhelming their already compromised reserve capacity.

The study also has broader implications for geroscience. Alcohol is shown here to be a powerful modulator of key aging pathways—mitochondrial dysfunction, oxidative stress, inflammation, cell death, and senescence. This positions alcohol not only as a toxicant but also as *an accelerator of biological aging*. This conclusion should stimulate further investigations into how other lifestyle factors and environmental exposures might interact with intrinsic aging processes to determine health span and resilience.

In conclusion, this study by Mukhopadhyay et al. [5] significantly advances our understanding of how alcohol consumption affects cardiovascular aging. The findings reported in this paper convincingly demonstrate that chronic alcohol intake accelerates the biological aging of the heart and vasculature, reduces cardiovascular reserve, and increases the risk of heart failure. Given the widespread prevalence of alcohol use and the global trend toward an aging population, these findings are both timely and clinically significant and should serve as a call on clinicians, researchers, and public health professionals to recognize alcohol use as a modifiable risk factor in the aging cardiovascular system.
